# Mechanoluminescent-Triboelectric Bimodal Sensors for Self-Powered Sensing and Intelligent Control

**DOI:** 10.1007/s40820-023-01054-0

**Published:** 2023-03-24

**Authors:** Bo Zhou, Jize Liu, Xin Huang, Xiaoyan Qiu, Xin Yang, Hong Shao, Changyu Tang, Xinxing Zhang

**Affiliations:** 1 State Key Laboratory of Polymer Materials Engineering, Polymer Research Institute, Sichuan University, Chengdu, 610065 People’s Republic of China; 2 Chengdu Development Center of Science and Technology, China Academy of Engineering Physics, Chengdu, 610200 People’s Republic of China

**Keywords:** Bimodal sensors, Mechanoluminescence, Triboelectric nanogenerator, Intelligent control, Self-powered

## Abstract

Self-powered flexible devices with skin-like multiple sensing ability have attracted great attentions due to their broad applications in the Internet of Things (IoT). Various methods have been proposed to enhance mechano-optic or electric performance of the flexible devices; however, it remains challenging to realize the display and accurate recognition of motion trajectories for intelligent control. Here, we present a fully self-powered mechanoluminescent-triboelectric bimodal sensor based on micro-nanostructured mechanoluminescent elastomer, which can patterned-display the force trajectories. The deformable liquid metals used as stretchable electrode make the stress transfer stable through overall device to achieve outstanding mechanoluminescence (with a gray value of 107 under a stimulus force as low as 0.3 N and more than 2000 cycles reproducibility). Moreover, a microstructured surface is constructed which endows the resulted composite with significantly improved triboelectric performances (voltage increases from 8 to 24 V). Based on the excellent bimodal sensing performances and durability of the obtained composite, a highly reliable intelligent control system by machine learning has been developed for controlling trolley, providing an approach for advanced visual interaction devices and smart wearable electronics in the future IoT era.

**Supplementary Information:**

The online version contains supplementary material available at 10.1007/s40820-023-01054-0.

## Highlights

- A fully self-powered bimodal sensor is designed for patterned-displaying the force trajectories.
- Outstanding mechanoluminescence is achieved with a stimulation force as low as 0.3 N and 2000 cycles reproducibility.
- The designed bimodal sensor exhibits good potential for handwriting input to achieve visual intelligent control.

**Supplementary Information:**

The online version contains supplementary material available at 10.1007/s40820-023-01054-0.

## Graphical abstract

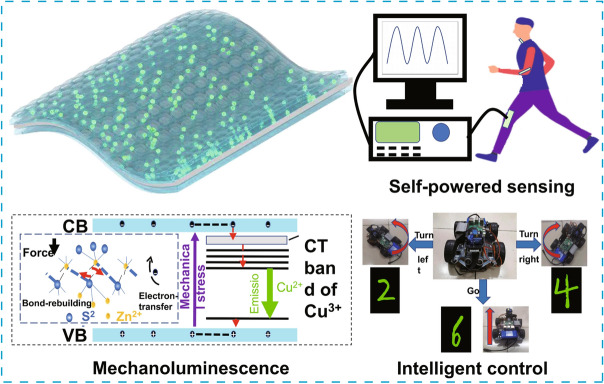

## Introduction

In nature, biological skin has attracted extraordinary attention due to its fascinating properties of stretchability [1–5], self-healing [6, 7] and multimodal sensing ability [8–12]. Biological skin allows organisms to interact with their surroundings and sense changes in external stimuli such as temperature [13–16], pressure [13, 17, 18] and pain. It transmits stimulus information to the brain via nerve conduction, enabling the organisms to respond and control. Especially when exposed to external environmental stimuli, some biological skin can present fluorescent or color responses for hazard avoidance, camouflage, and courtship. For example, once cephalopods are under dangerous situations, they can fluoresce to avoid danger by the muscle-controlled movements of chromatophores filled with pigment sacs [19, 20]. Inspired by the biological skin, mechanoluminescent materials are being utilized in the fabrication of electronic skin (e-skin) for increasing the functionality and identifiability of wearable electronics [21, 22].

Mechanoluminescence (ML) material can establishes a link between mechanical and optical signals for mechanical visual sensing. Generally, the functional materials are prepared by chemical synthesis [23, 24] and nano-microstructure strategies [25, 26]. While the former approach usually relies on specific force-sensitive chromophore molecules (e.g., spiropyran based on force-induced breakage of C–N and C–O bonds) [27], the latter one can reflect natural light through elaborate structures to produce distinct color alternations and fluorescent changes. Craig and his colleagues reported ML e-skin with improved mechanical sensitivity by constructing multilayer nanoparticle microporous structures in ML polymers [22]. Priya et al. introduced a sulfur vacancy in the zinc sulfide doped with copper (ZnS:Cu) phosphors, which is able to induce the formation of new energy levels to improve the luminescence performance [28]. However, most work focused on enhancing ML intensity and achieving the display and accurate recognition of motion trajectories based on ML remains a great challenge.

Mimicking the multimodal sensing ability of animal skin is another target for next-generation e-skin materials [29–31]. Bao and colleagues have developed an e-skin capable of interactive color change and haptic sensing properties based on stretchable resistive pressure sensors and electrochromic devices [32]. Zhang and colleagues demonstrated a pressure–temperature bimodal tactile sensor by combining fundamentally different sensing mechanisms of optical and electronic devices, thus enabling simultaneous independent sensing of pressure and temperature [33]. However, the achievement of multimodal sensing usually requires external energy input and additional devices for the successful operation, which limits their practical applications [30, 33, 34]. The self-powered, multimodal and visualized sensor for intelligent control is urgently needed in the era of Internet of Things (IoT) and fifth-generation wireless networks [35–37].

In this work, we demonstrate a mechanoluminescent-triboelectric bimodal sensor (MTBS) that intuitively detects force signals and enables both intelligent control and human physiological activity detection through force-optical and force-electric response. The ZnS:Cu particles are incorporated into polydimethylsiloxane (PDMS) elastomer to generate ML properties, and meanwhile corresponding software is developed to convert transient luminescence into visual images for intelligent control based on interdisciplinary machine learning algorithms. Furthermore, the stress transfer is stabilized by introducing liquid metals (LMs) with excellent electrical conductivity; thereby, the entire device exhibits outstanding mechanoluminescence. The surface microstructures built on the ML materials achieves excellent output of triboelectric nanogenerator (TENG) for power supply of the sensor. This bioinspired nano-microstructured bimodal sensor provides a new solution for preparing fully self-powered visualized multimodal sensing systems, showing potential applications in wearable electronic devices and human–computer interaction.

## Experimental Section

### Materials

ZnS:Cu was purchased from Shanghai Keyan Photoelectric Technology Co., Ltd (China). Liquid vinyl-terminated polydimethylsiloxane (PDMS) (Sylgard184, Part A) with a curing agent (Part B) was supplied by Dow Chemical Company (USA). Gallium (Ga, > 99.99%) and Indium (In, > 99.99%) were obtained from Shanghai Aladdin Biological Technology Co., Ltd (China). The water was deionized and ultrafiltered to 18.2 MΩ cm^−1^ with an ultrapure water system (China).

### Fabrication of the Bimodal Sensors

The mechanoluminescent layer was prepared by following steps. ZnS:Cu particles were mixed with PDMS (Part A) in the ratio of 7:3 and formed a uniform dispersion followed by adding the curing agent (Part B) according to a PDMS/curing agent ratio of 10:1. Then, the mixture was poured into a PTFE mold with a layer of sandpaper and was cured at 80 °C for 2 h. Then, EGaIn alloys were prepared by heating Ga/In (3:1 mass ratio) mixture in a nitrogen atmosphere at 120 °C for 2 h. The resulted EGaIn alloys (liquid metal) were dropped onto PDMS/ZnS:Cu composite elastomer to build the electrodes, and then copper foil was pasted onto their surfaces for electrical measurements. Finally, PDMS was uniformly coated and was cured on the liquid metal for preventing leakage of the liquid metal.

### Preparation of Tests Sample for Fluorescence Spectroscopic

The composite elastomers were charged manually through friction-separation for several times with PET. To eliminate the influence of charged electrons on ML performance, all samples were blown by an ion fan for 30 min to remove charges, and then, fluorescence spectroscopic characterization was carried out again.

### Characterization

The surface structure of PDMS/ZnS:Cu and the distribution of ZnS:Cu in the cross section were obtained from SEM (JSM-5600, JEOL, Japan). Energy-dispersive spectrometer was used to determine the element distribution in ZnS: Cu (Octane Elect Super, EDAX, USA). XRD patterns were recorded from 10° to 70° (scanning rate 10° min^–1^) using an Ultima IV instrument (Rigaku, Japan) equipped with Cu-Ka radiation (*λ* = 0.1540 nm). Mechanoluminescence properties tests were carried out on a universal tensile testing machine (Instron-5966, USA) with a cross-head speed of 100–1000 mm/min^–1^ at room temperature. The triboelectric performance of MTBS was measured with a linear motor (HS01–37 166, NTI AG, USA), an electrostatic tester (6514, Keithley, USA) and an amplifier (SR570 type, SRS, USA). The fluorescence spectroscopy was performed on FluoroMax-4 (HORIBA, Japan) to characterize the luminous intensity. All the mechanoluminescent images in this paper were captured with Huawei mate40.

## Result and Discussion

### Materials Design

**Fig. 1:**
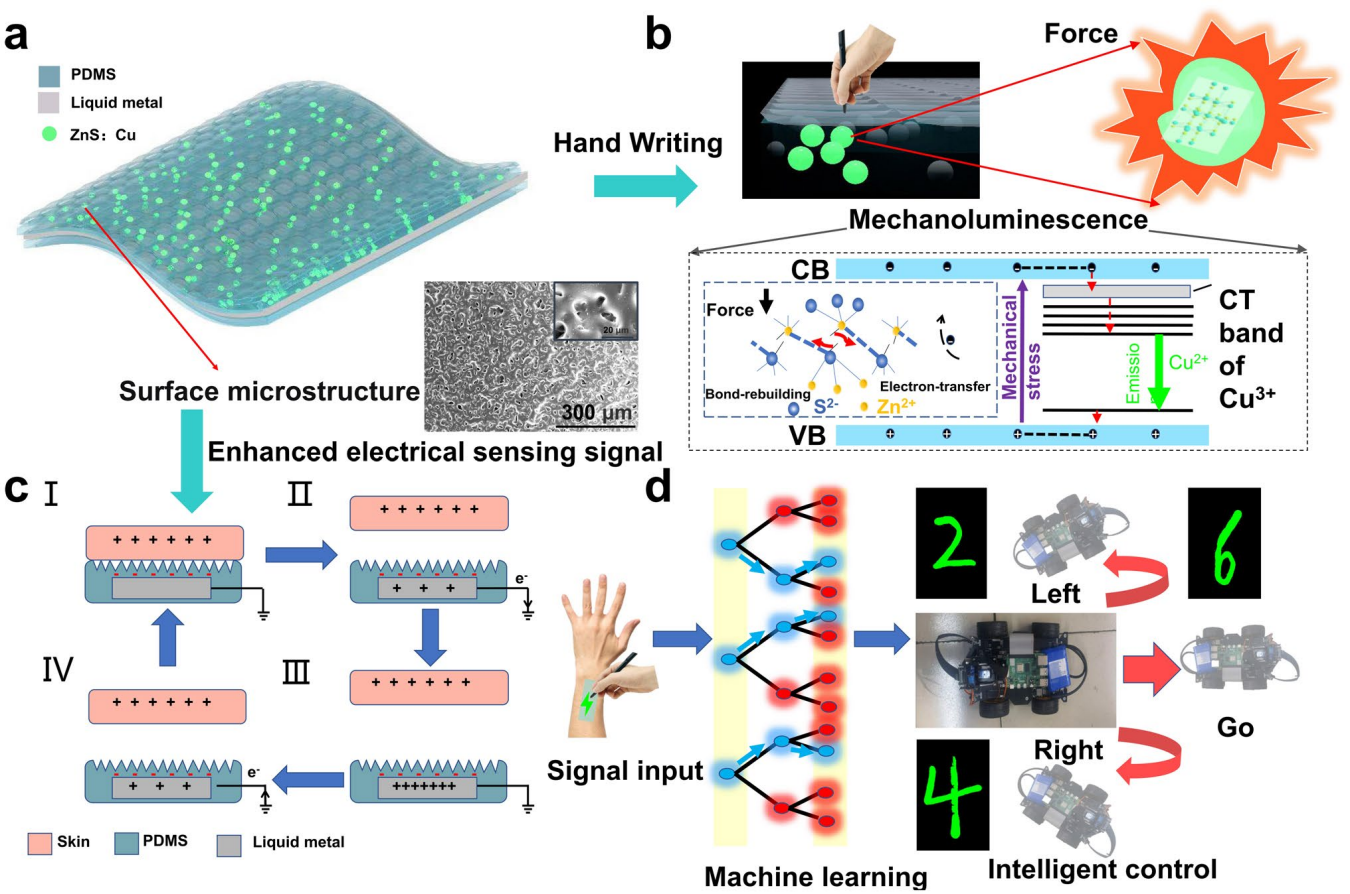
Structure design of the MTBS. **a** Schematic of the design for the bimodal self-powered sensor. **b** ML mechanism of ZnS:Cu particles. **c** Schematic diagrams of the working principle of the MTBS. **d** Developed intelligent control system by recognizing handwriting numbers based on interdisciplinary machine learning approach

As shown in Fig. 1a, benefit from its multilayer structure design, the MTBS possesses an optoelectronic dual-signal sensing mode with unique characteristics. Specifically, one single stimulus is able to produce both electrical and optical signals at the same time, which is only drove by mechanical forces (no external power supply is required). The MTBS is a typical layer-by-layer structure which consists of three parts from top to bottom based on the contact-separation mode TENG [38]. The top layer is ML elastomer consisting of PDMS with well-dispersed ZnS:Cu particles, which can produce optical signal under external force. Moreover, the introduction of sandpaper-generated surface microstructures is able to enhance the charge density on the triboelectric surface and improve the sensitivity of sensing detection [39]. When the obtained composite elastomer subjected to external forces, the defects (point, line and planar defects) of ZnS crystals start to move, which leads to the breakage and reconstruction of Zn–S chemical bond (Fig. 1b). The covalently bonded *s* and *p* electrons will be redistributed after a Zn–S bond breaking, leading to an energy band bending and the tunneling of trapped electrons to the conduction band. Some of the de-trapped electrons move into the conduction band and recombine with holes. Thus, the released energy during the electron–hole complex excites the doped Cu^2+^ ions and then the de-excitation of excited Cu^2+^ ions lead to a luminescence [40, 41]. In the middle part, gallium-based LMs are selected as stretchable electrodes due to their low melting points, low viscosity, high conductivity and non-toxicity [42–45]. Meanwhile, their fluidity makes the stress transfer more stable through overall device to ensure ML performance. The lower part made of PDMS is used as a triboelectric and encapsulation layer to prevent leakage of the electrodes. It is worth noting that LMs are able to form a dense oxide layer on the surface immediately after contact with air during usage, which is effective enough to prevent further oxidation and limit the penetration into PDMS during prolonged stretching [46, 47]. Therefore, the oxide layer of LMs makes the electrical signals of the MTBS more stable and durable.

MTBS acts on the coupling of triboelectrification and electrostatic induction, where the skin and PDMS are considered as positive and negative frictional electric material, respectively. As shown in Fig. 1c, human skin acts as the ground and the MTBS attached to human body acts as a single electrode TENG. Since silicone rubber possesses a strong ability to gain electrons while human skin with a strong tendency to lose electrons, silicone rubber is well suited to be a triboelectric layer material for self-powered human motion sensing [48]. During the cyclic contact and separation between skin and the MTBS, alternating currents can be generated due to the flow of charges. Based on this visualized bimodal sensor, various human–machine interface scenarios are demonstrated, including intelligent control with high recognition rate through machine learning and human physiological activity monitoring (Fig. 1d).

### Mechanoluminescent Sensing

**Fig. 2:**
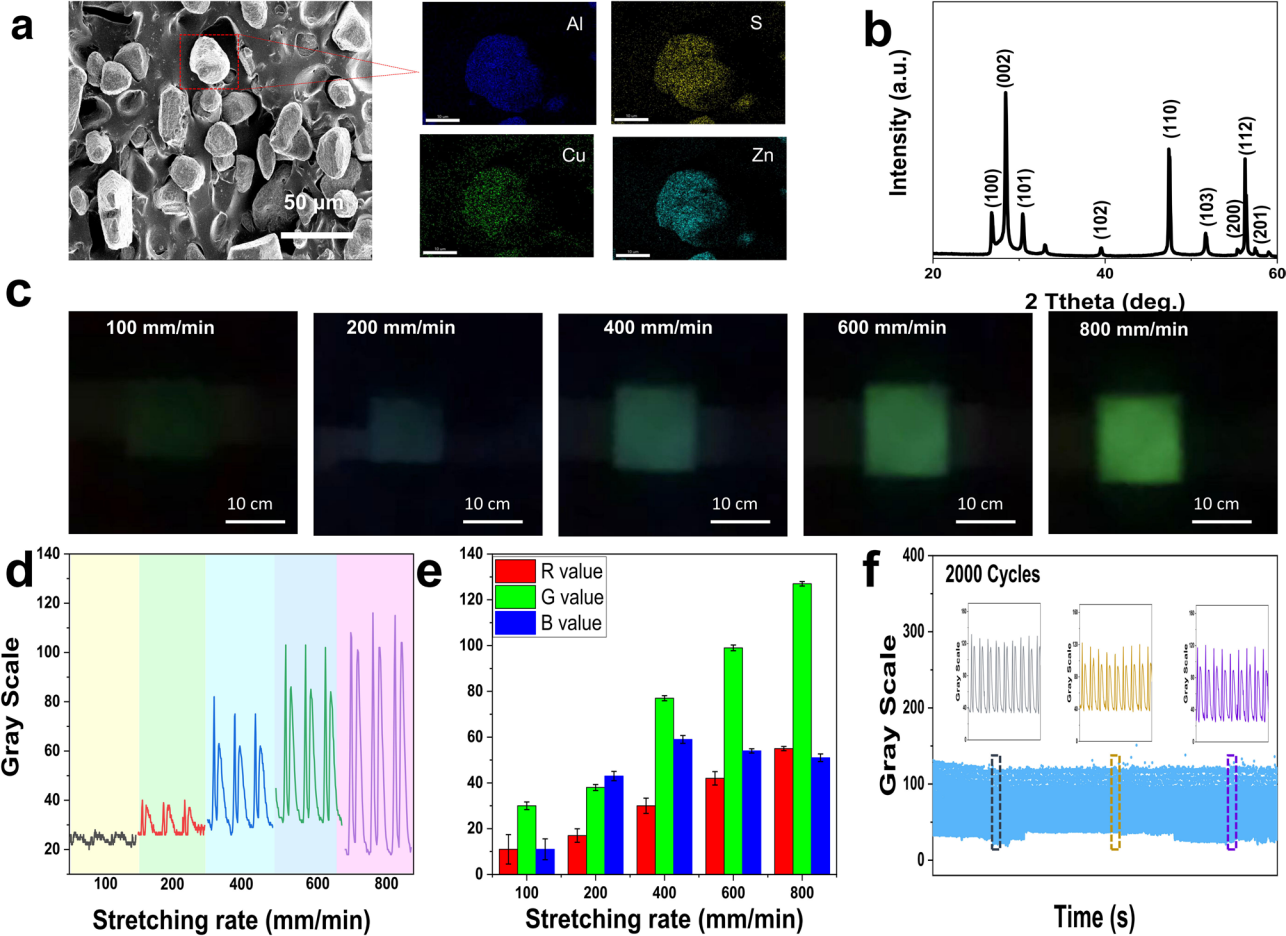
ML output performance of the composite elastomer. **a** Cross-sectional SEM images of PDMS/ZnS:Cu composite elastomer and EDS mapping of individual ZnS:Cu particles. **b** XRD patterns of ZnS:Cu particles. **c** Digital images of mechanoluminescent elastomer at a range of stretch rates. **d** Changes in grayscale values of mechanoluminescent elastomer at a range of stretch rates. **e** RGB values of mechanoluminescent elastomer vary with the stretching rate. **f** Durability of mechanoluminescent elastomer for more than 2000 stretching-releasing cycles

The ML layer of the visualized bimodal sensor is achieved by homogeneously embedding the ZnS:Cu phosphors into PDMS (PDMS/ZnS:Cu). According to the cross-sectional scanning electron microscopy (SEM) of the PDMS/ZnS:Cu composite elastomer shown in Fig. 2a, ZnS:Cu particles with an average particle size of around 20 μm are uniformly embedded in PDMS matrix. Energy-dispersive X-ray (EDX) spectral mapping reveals the elemental distribution of individual ZnS:Cu particles. In addition to the elements Zn, S and Cu, there is also Al on the surface for moisture resistance. X-ray powder diffraction (XRD) confirms the presence of the wurtzite structure of ZnS:Cu phosphors (Fig. 2b) [49]. The changes in brightness and color during force luminescence were analyzed according to the standards of the International Commission on Luminescence. The ML performances of the composite elastomer under stretching (30% strain) are shown in Fig. 2c. The luminescence intensity of the composite elastomer increases with increasing stretching rate (from 100 to 800 mm min^−1^). Grayscale color analysis is used as an efficient and scientific method to accurately indicate the lightness/darkness of the pixel in images of the composite elastomer [50–52]. As shown in Fig. 2c–d, both the intensity and grayscale value of the luminescence increase with increasing stretching rate. The relationship between stretching rate and grayscale values is approximately linear with a goodness of fit > 97% (Fig. S1). In addition, color variations are quantified by RGB color intensity analysis. RGB is the color representing the three channels of red (*R*), green (*G*) and blue (*B*), and all colors are obtained by the variations of the three RGB color channels and their superposition on each other [53, 54]. We observe that the *R* and *B* values of elastomer luminescence did not change much with increasing stretching rate, while the *G* value changes sharply (Fig. 2e) and linearly (Fig. S2). This color change characteristic lays the foundation for the subsequent signal collection and processing. The stress–strain curves of the composite elastomer are shown in Fig. S3. The results show that the composite material has good mechanical properties and can meet the needs of human motion monitoring. To test the durability and stability of the ML performances of the obtained MTBS, the PDMS/ZnS:Cu composite elastomer is subjected to 2000 times of stretch-released cycles by stretching at a strain of 30% (Fig. S4). As shown in Fig. 2f, the repeated stretching-releasing processes have almost no effect on the ML performances of the composite elastomer, which is crucial for the sensing of the elastomer in practical visualization. Excellent repeatability of ML is important for multiple writing, intelligent recognition stability, etc. More importantly, the light intensity increases with the number of frictions (Fig. S5). Because the triboelectric effect supplies an external field that can reduce the trap-depth of charges in ZnS:Cu. More trapped charges, especially the deep-trapped charges would be released by the in situ internal piezoelectric field of ZnS:Cu, resulted in the improved luminescence property.

### Triboelectric Sensing

**Fig. 3:**
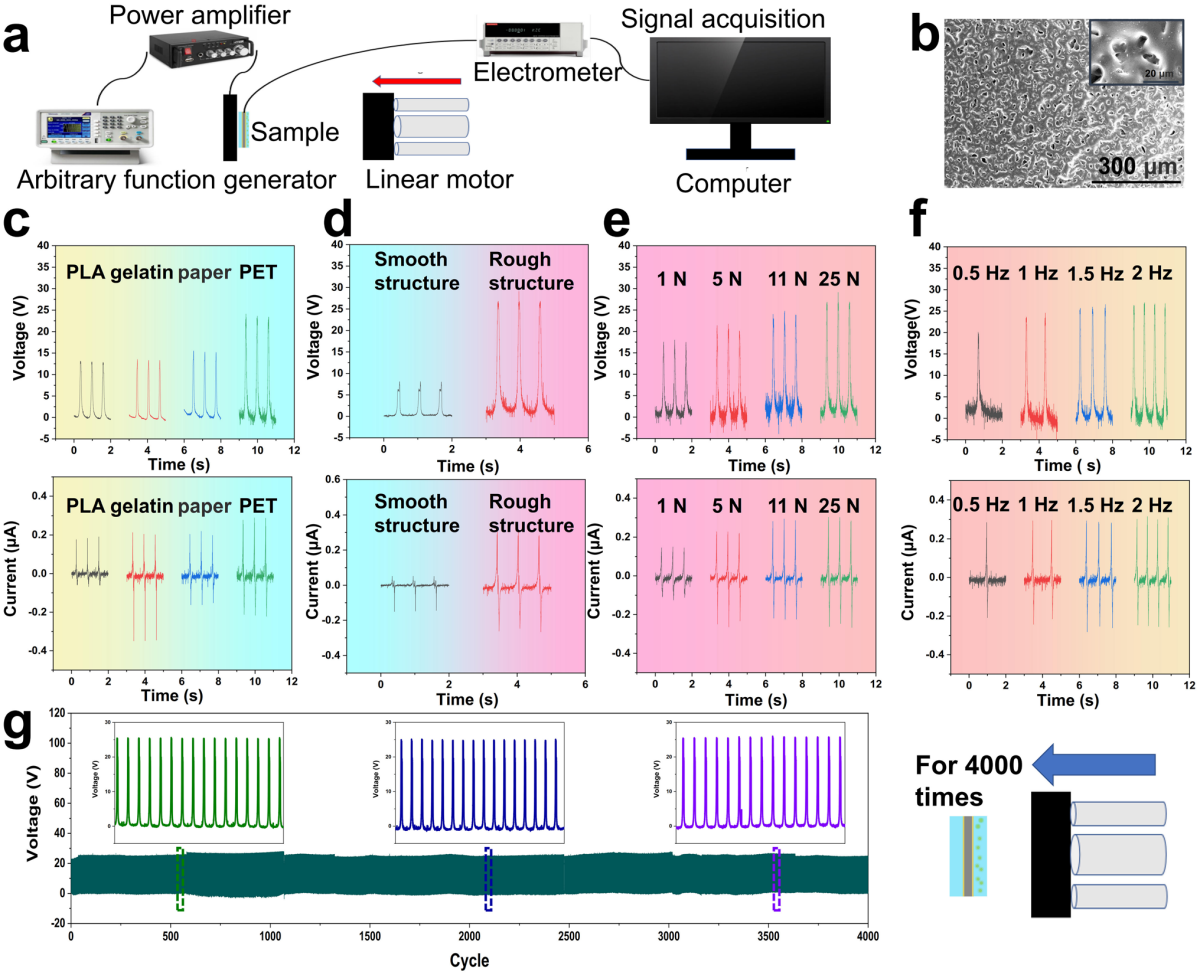
Electrical output performance of the MTBS.** a** schematic of TENG test system. **b** SEM images of the microstructures on the surface of PDMS/ZnS:Cu layer. **c** Output voltage and current of the MTBS in contact with different cathode materials. **d** MTBS output voltage and current for smooth and rough surfaces. **e** MTBS output voltage and current under different contact forces. **f** MTBS output voltage and current under different frequencies. **g** Long-term stability of MTBS output under 4000 loading–unloading cycles

MTBS possesses excellent ML performance and good self-powered TENG performance, which can be used in collecting mechanical energy in different environments and is able to realize a self-powered sensing without battery [55–58]. The output performances of the MTBS were tested by a home-made measurement system consisting of a function generator, a power amplifier, a linear motor, an electrostatic meter, and a signal acquisition computer [36, 59] (Fig. 3a). In order to study the effect of different contact materials on the output performance of TENG, polylactic acid (PLA), gelatin, paper and polyethylene terephthalate (PET) were used as friction materials and the open-circuit voltage (*V*_oc_) and short-circuit current (*I*_sc_) were measured (Fig. 3c). Among these friction materials, both the *V*_oc_ and *I*_sc_ values reach a maximum in PET, due to the maximum difference in electron gain and loss between PET and PDMS/ZnS:Cu composite films. In subsequent tests, PET was selected as the friction material. To improve the sensitivity, we construct microstructures on the surface of PDMS/ZnS:Cu layer **(**Fig. 3b). Figure 3d shows the effect of structured surface on the *V*_oc_ and *I*_sc_. The smooth and rough surfaces were compared separately (rough surface was obtained by sandpaper molds while smooth surface was obtained by smooth Teflon molds). The voltage of devices with smooth surface is only about 8 V, while the voltage of devices with rough surface increases to 24 V (a three-fold increase). Generally, the output of TENG under contact separation mode is highly dependent on the applied pressure and the frequency of contact [17, 60]. Under the normal impact force, the device can generate different *V*_oc_ and *I*_sc_ and the normal force range is 1–25 N. Both the *V*_oc_ and *I*_sc_ increase with the increase in impact force, which can reach a maximum *V*_oc_ of 27 V and *I*_sc_ of 0.3 μA under 25 N of applied normal impact force (Fig. 3e). When the frequency of the applied force increased from 0.5 to 2 Hz, the current is around 0.3 μA, but it still increases slightly. The *V*_oc_ increased from 20 to 27 V from 0.5 to 2 Hz (Fig. 3f). The MTBS also possesses a very fast response and recovery speed of only 61 and 70 ms after applying or releasing, which makes it possible to monitor external stimuli in real time (Fig. S6). The use of the obtained MTBS as a sensor for human motion monitoring means that it may be subjected to repeated mechanical forces and shocks during usage, so the durability and durability of the devices is very important. As shown in Fig. 3g, after 4000 cycles of applied force (5 N), there is no significant drop of output signals of the device. In summary, these results show that the obtained MTBS possesses significant advantages in the practical applications of self-powered sensing.

**Fig. 4:**
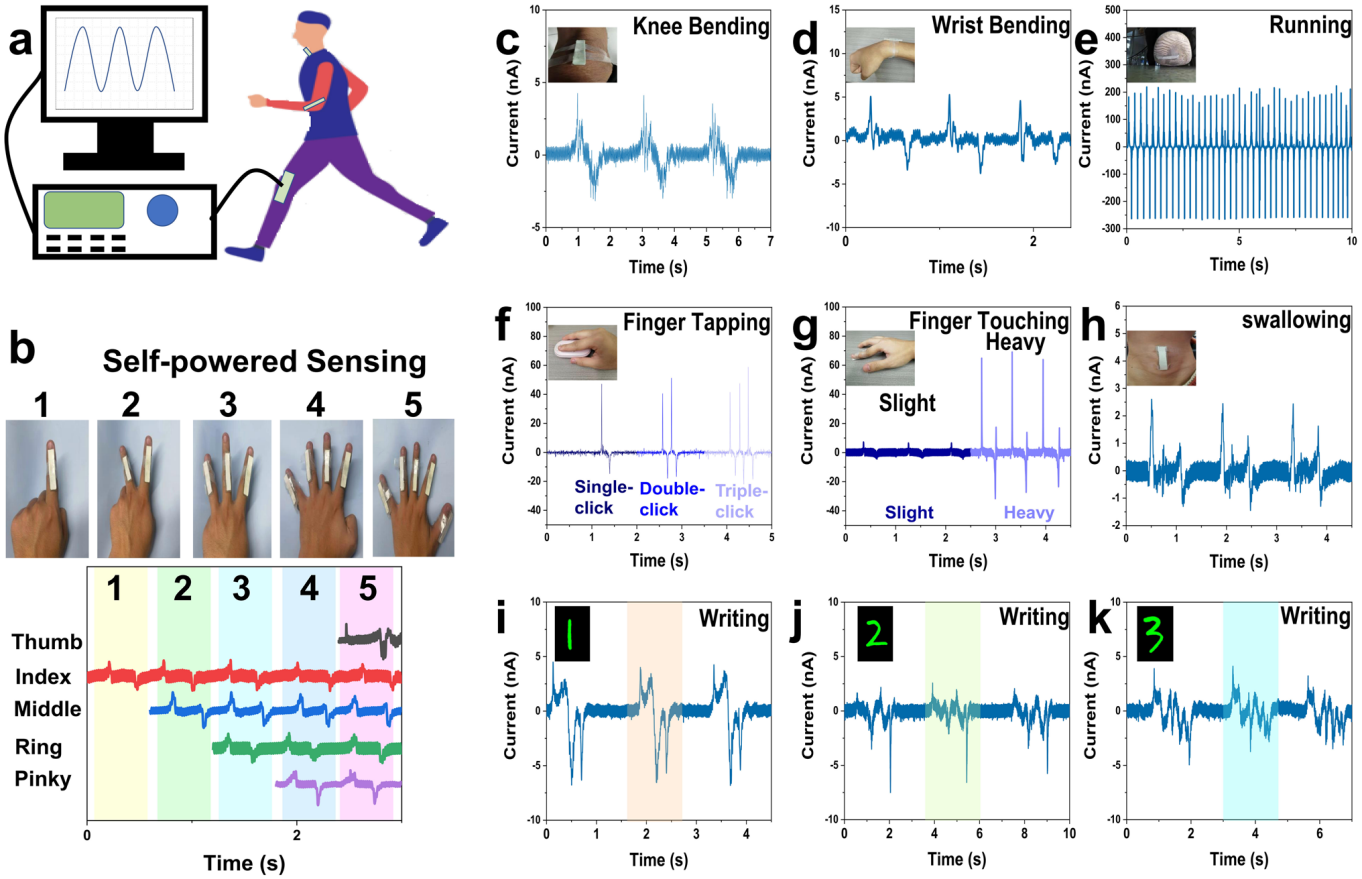
Self-powered sensor performance of the MTBS.** a** Demonstration of the system for monitoring human physiological signals based on the obtained MTBS. Signals of the MTBS used as self-powered sensors to monitor **b** Gestures recognizing, **c** knee bending, **d** wrist bending, **e** running, **f** finger tapping, **g** finger touching, **h** swallowing, **i** writing Arabic number “1”, **j** writing Arabic number “2”, **k** writing Arabic number “3”

To demonstrate the capability of the obtained MTBS as a self-powered physiological sensor, several practical applications for its dynamic mapping of human motion and writing are performed separately **(**Fig. 4a). As shown in Fig. 4c–d, the MTBS was attached to the volunteer’s wrist and knee, and the sensor is able to immediately responds by outputting signals according to the body movement. In addition, the MTBS can also be used as a pedometer for monitoring human movements, based on the fact that each movement with contact-separation action is able to produce a clear output signal (~ 200 nA**,** Fig. 4e). Then, the MTBS was mounted on the mouse to differentiate frequencies of mouse clicks (Fig. 4f). By using the MTBS-attached finger to touch the table in different strength, there is a large difference of the output signals (~ 8 nA vs. 60 nA, Fig. 4g). The designed MTBS can detect larger-scale human movements as well as subtle human movements. As shown in Fig. 4h, when we attached the MTBS to the volunteer's throat the sensor can generate regular current signals as the vibration of the throat. In addition, wearable gesture sensors capable of sensing dexterous movements of each finger can play an important role in future human–computer interaction interfaces. As shown in Fig. 4b, when the volunteer expresses the Arabic numbers 1, 2, 3, 4 and 5 by gestures, the sensor with different signal combination states (every finger movement can be detected separately) can be used to accurately identify the meaning of different gestures. As shown in Fig. 4i–k, when different numbers are written on the MTBS using a pen, there are different current signal outputs as well as visual pictures of the numbers. Since the luminescent signals generated by the MTBS during handwriting are transient for about 200 ms (Video S1 and Fig. S7), which is almost impossible to accomplish for recognition. A tool has been developed to obtain clear digital images by continuously intercepting multiple frames from the input video and combining them for superimposition. Take the writing of number “2” as an example, by using a pen to write on the composite elastomer and intercepting the consecutive images (Fig. S8), the trajectory of the force applied to the material can be clearly seen, which is important for the visual monitoring of writing process on the obtained composite elastomer. Overall, the MTBS shows excellent stability and sensitivity for human motion recognition and possesses great potential for future applications in wearable electronics. Further, the possibility of bright handwriting on MTBS based on the desired ML properties can expand the scope of its applications.

### Signal Identification and Intelligent Control

**Fig. 5:**
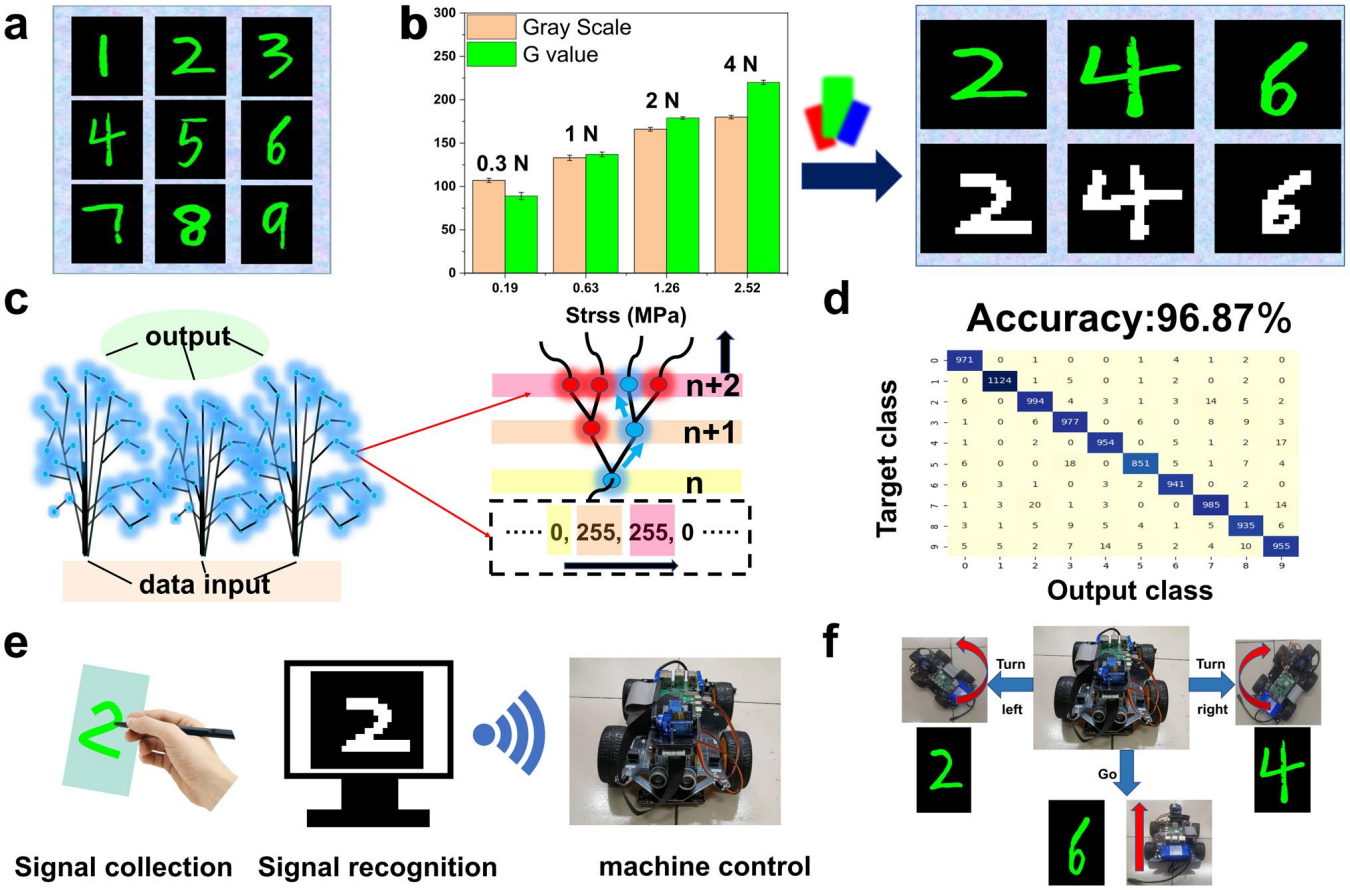
Intelligent control system. **a** Images of Arabic numeral from 1 to 9 processed by the developed software. **b** Histogram of Gray Scale and *B*-value in response to the applied force during handwriting and the conversion of generated images to the images with white character with black background grayscale for Arabic numbers 2, 4 and 6. **c** Schematic diagram of RF machine learning algorithm. **d** Prediction confusion matrix based on 10,000 test data. **e** Schematic diagram of handwriting numerically controlled trolley movement system. **f** Movement of the trolley is controlled by different input digital signals, such as left turn, right turn and straight ahead. (Color figure online)

Based on the excellent performances of the obtained MTBS, the clear-cut and bright images for controllable handwriting is acquired (Fig. 5a), which greatly facilitate the accurate recognition and efficient intelligent control. The frame-by-frame enhancement magnification can be adjusted to improve some of the digital defects during the image composition process (Fig. S9). In addition, to facilitate the recognition process based on machine learning approach, the training process of the machine learning network was performed based on the Mixed National Institute of Standards and Technology database (MNIST), an open-source third-party database of handwriting digits [61, 62]. In order to improve recognition efficiency and accuracy, the handwriting digital images based on the obtained MTBS (extracted green color at first to avoid disturbance) and then converted to the digitization matrix (Fig. S10). As shown in Fig. S11, compared with extracting red and blue, extracting green for image synthesis makes the writing content the clearest. Consist with the above characterization about ML performances, the grayscale and G-value of the writing area change significantly with the applied force and a very small force is enough for the achievement of handwriting signal input (Fig. 5b). The MTBS can be used to distinguish the obtained handwriting patterns and has great potential for intelligent control by recognizing numbers.

Machine learning can be used for handwritten digit recognition through various algorithms [63, 64]. Random forest (RF) algorithm, which integrates multiple decision trees through the idea of integrated learning, was used to recognize the handwriting digital images [65–67]. The correct recognition rate of the three algorithms was compared (random forest, *K*-nearest neighbor, decision tree), the random forest algorithm has the highest correct rate because it integrates multiple decision trees through the idea of integrated learning (Fig. S12). RF algorithm is to determine the result by category voting after classifying the input data according to their different features, and designate the category with the highest number of votes as the final output result (Fig. 5c). The correct rates of RF algorithm-based machine learning with training amounts of 1000, 5000, 10,000, 30,000 and 60,000 are compared, and it is found that the correct rate of machine learning increases as the training amount increases. However, the time required for machine learning also increases, so in order to balance the time required for machine learning with the correct rate, we choose the training amount of 60,000 for identification (Fig. S13). The developed RF algorithm-based machine learning network was trained by using 60,000 handwriting digits from the open-source MNIST handwriting digit database (Fig. S14), and the accuracy was later verified by using another 10,000 handwriting digits for test. The results show that the model used for recognizing hand-written digits possesses a high accuracy rate (96.87%, Fig. 5d), verifying the good reliability of digit recognition system based on machine learning. To further validate this concept, videos of writing 2, 4 and 6 on the MTBS were imported into the developed integrated software (combing data reading, image synthesis, recognition and control), after which the handwriting information recognized by the software is transmitted to the trolley via wireless signals to achieve intelligent remote control of the trolley **(**Fig. 5e). Figure 5f and Videos S2–S4 show that by defining the moving mode, the trolley can move forward and turn left/right according to the written numbers **(**Video S5). This handwriting-controlled motion system can be used to manipulate machines to help humans perform complex operations and has great promise for intelligent control and human–machine interface in the future.

## Conclusion

In summary, we propose a visualized bimodal sensor which is easy to be manufactured and completely self-powered. Through the design of a multilayer structure based on deformable LMs combined with a micro-nano-structured mechanoluminescent elastomer, the obtained MTBS can output synergistic optoelectronic signals under stress with good reproducibility. In addition, thanks to the processing of transient optical signals, visualization of handwriting and intelligent control by machine learning are achieved. We also demonstrate the application of the device in gesture recognition. Our research provides ideas for future bimodal sensing mechanisms that have broad application scenarios in intelligent control and visual interaction devices.

### Supplementary Information

Below is the link to the electronic supplementary material.

Supplementary file1 (MP4 58 KB)

Supplementary file2 (MP4 6907 KB)

Supplementary file3 (MP4 6919 KB)

Supplementary file4 (MP4 7164 KB)

Supplementary file5 (MP4 26742 KB)

Supplementary file6 (PDF 974 KB)

## References

[1] Wang S, Xu J, Wang W, Wang GN, Rastak R (2018). Skin electronics from scalable fabrication of an intrinsically stretchable transistor array. Nature.

[2] Jung D, Lim C, Shim HJ, Kim Y, Park C (2021). Highly conductive and elastic nanomembrane for skin electronics. Science.

[3] Rao Z, Thukral A, Yang P, Lu Y, Shim H (2022). All-polymer based stretchable rubbery electronics and sensors. Adv. Funct. Mater..

[4] Yu Y, Yi P, Xu W, Sun X, Deng G (2022). Environmentally tough and stretchable mxene organohydrogel with exceptionally enhanced electromagnetic interference shielding performances. Nano-Micro Lett..

[5] Liu J, Guo Q, Mao S, Chen Z, Zhang X (2018). Templated synthesis of a 1D Ag nanohybrid in the solid state and its organized network for strain-sensing applications. J. Mater. Chem. C.

[6] Wang Y, Huang X, Zhang X (2021). Ultrarobust, tough and highly stretchable self-healing materials based on cartilage-inspired noncovalent assembly nanostructure. Nat. Commun..

[7] Ding Q, Zhou Z, Wang H, Wu Z, Tao K (2022). Self-healable, recyclable, ultrastretchable, and high-performance NO_2_ sensors based on an organohydrogel for room and sub-zero temperature and wireless operation. SmartMat.

[8] Lou Z, Chen S, Wang L, Shi R, Li L (2017). Ultrasensitive and ultraflexible e-skins with dual functionalities for wearable electronics. Nano Energy.

[9] Zhang H, Chen H, Lee JH, Kim E, Chan KY (2022). Bioinspired chromotropic ionic skin with in-plane strain/temperature/pressure multimodal sensing and ultrahigh stimuli discriminability. Adv. Funct. Mater..

[10] Xie M, Hisano K, Zhu M, Toyoshi T, Pan M (2019). Flexible multifunctional sensors for wearable and robotic applications. Adv. Mater. Technol..

[11] Li WD, Ke K, Jia J, Pu JH, Zhao X (2022). Recent advances in multiresponsive flexible sensors towards e-skin: a delicate design for versatile sensing. Small.

[12] Cao W, Wang Z, Liu X, Zhou Z, Zhang Y (2022). Bioinspired mxene-based user-interactive electronic skin for digital and visual dual-channel sensing. Nano-Micro Lett..

[13] Kim SY, Park S, Park HW, Park DH, Jeong Y (2015). Highly sensitive and multimodal all-carbon skin sensors capable of simultaneously detecting tactile and biological stimuli. Adv. Mater..

[14] Xiong W, Zhu C, Guo D, Hou C, Yang Z (2021). Bio-inspired, intelligent flexible sensing skin for multifunctional flying perception. Nano Energy.

[15] Yue O, Wang X, Liu X, Hou M, Zheng M (2021). Spider-web and ant-tentacle doubly bio-inspired multifunctional self-powered electronic skin with hierarchical nanostructure. Adv. Sci..

[16] Li X, Liu J, Li D, Huang S, Huang K (2021). Bioinspired multi-stimuli responsive actuators with synergistic color- and morphing-change abilities. Adv. Sci..

[17] Yi J, Dong K, Shen S, Jiang Y, Peng X (2021). Fully fabric-based triboelectric nanogenerators as self-powered human–machine interactive keyboards. Nano-Micro Lett..

[18] Zhu P, Wang Y, Wang Y, Mao H, Zhang Q (2020). Flexible 3d architectured piezo/thermoelectric bimodal tactile sensor array for e-skin application. Adv. Energy Mater..

[19] Guo Q, Huang B, Lu C, Zhou T, Su G (2019). A cephalopod-inspired mechanoluminescence material with skin-like self-healing and sensing properties. Mater. Horiz..

[20] Zeng S, Zhang D, Huang W, Wang Z, Freire SG (2016). Bio-inspired sensitive and reversible mechanochromisms via strain-dependent cracks and folds. Nat. Commun..

[21] Sun Y, Wang Y, Liu Y, Wu S, Zhang S (2022). Biomimetic chromotropic photonic-ionic skin with robust resilience, adhesion, and stability. Adv. Funct. Mater..

[22] Park J, Lee Y, Barbee MH, Cho S, Cho S (2019). A hierarchical nanoparticle-in-micropore architecture for enhanced mechanosensitivity and stretchability in mechanochromic electronic skins. Adv. Mater..

[23] Humeniuk HV, Rosspeintner A, Licari G, Kilin V, Bonacina L (2018). White-fluorescent dual-emission mechanosensitive membrane probes that function by bending rather than twisting. Angew. Chem. Int. Ed..

[24] Zhang L, Shi K, Wang Y, Su L, Yang G (2021). Unraveling the anomalous mechanoluminescence intensity change and pressure-induced red-shift for manganese-doped zinc sulfide. Nano Energy.

[25] Ito MM, Gibbons AH, Qin D, Yamamoto D, Jiang H (2019). Structural colour using organized microfibrillation in glassy polymer films. Nature.

[26] Li X, Liu J, Guo Q, Zhang X, Tian M (2022). Polymerizable deep eutectic solvent-based skin-like elastomers with dynamic schemochrome and self-healing ability. Small.

[27] Kempe F, Brugner O, Buchheit H, Momm SN, Riehle F (2018). A simply synthesized, tough polyarylene with transient mechanochromic response. Angew. Chem. Int. Ed..

[28] Qi T, Xia H, Zhang Z, Kong S, Peng W (2017). Improved water resistance of sral_2_O_4_: Eu^2+^, dy^3+^ phosphor directly achieved in a water-containing medium. Solid State Sci..

[29] Zhao J, Wei Z, Li Z, Yu J, Tang J (2021). Skin-inspired high-performance active-matrix circuitry for multimodal user-interaction. Adv. Funct. Mater..

[30] Lv Z, Liu J, Yang X, Fan D, Cao J (2020). Naturally derived wearable strain sensors with enhanced mechanical properties and high sensitivity. ACS Appl. Mater. Interfaces.

[31] Liu J, Zhao F, Tao Q, Cao J, Yu Y (2019). Visualized simulation for the nanostructure design of flexible strain sensors: from a numerical model to experimental verification. Mater. Horiz..

[32] Chou H-H, Nguyen A, Chortos A, To JW, Lu C (2015). A chameleon-inspired stretchable electronic skin with interactive colour changing controlled by tactile sensing. Nat. Commun..

[33] Ma X, Wang C, Wei R, He J, Li J (2022). Bimodal tactile sensor without signal fusion for user-interactive applications. ACS Nano.

[34] Pu J, Gao Y, Cao Q, Fu G, Chen X (2022). Vanadium metal-organic framework-derived multifunctional fibers for asymmetric supercapacitor, piezoresistive sensor, and electrochemical water splitting. SmartMat.

[35] Yu Z, Cai G, Liu X, Tang D (2021). Pressure-based biosensor integrated with a flexible pressure sensor and an electrochromic device for visual detection. Anal. Chem..

[36] Huang X, Wang Y, Zhang X (2022). Ultrarobust, hierarchically anisotropic structured piezoelectric nanogenerators for self-powered sensing. Nano Energy.

[37] Cai Y-W, Zhang X-N, Wang G-G, Li G-Z, Zhao D-Q (2021). A flexible ultra-sensitive triboelectric tactile sensor of wrinkled PDMS/MXene composite films for e-skin. Nano Energy.

[38] Zhao X, Wang Z, Liu Z, Yao S, Zhang J (2022). Anti-freezing and stretchable triboelectric nanogenerator based on liquid electrode for biomechanical sensing in extreme environment. Nano Energy.

[39] Pan R, Xuan W, Chen J, Dong S, Jin H (2018). Fully biodegradable triboelectric nanogenerators based on electrospun polylactic acid and nanostructured gelatin films. Nano Energy.

[40] Qasem A, Xiong P, Ma Z, Peng M, Yang Z (2021). Recent advances in mechanoluminescence of doped zinc sulfides. Laser Photonics Rev..

[41] Zhou H, Du Y, Wu C, Jiang Y, Wang F (2018). Understanding the mechanoluminescent mechanisms of manganese doped zinc sulfide based on load effects. J. Lumin..

[42] Liu J, Guo Q, Zhang X, Gai J, Zhang C (2021). Multistage responsive materials for real-time, reversible, and sustainable light-writing. Adv. Funct. Mater..

[43] Wang H, Chen S, Zhu X, Yuan B, Sun X (2022). Phase transition science and engineering of gallium-based liquid metal. Matter.

[44] Huang X, Liu J, Zhou P, Su G, Zhou T (2022). Ultrarobust photothermal materials via dynamic crosslinking for solar harvesting. Small.

[45] Yang X, Su G, Huang X, Liu J, Zhou T (2022). Noncovalent assembly enabled strong yet tough materials with room-temperature malleability and healability. ACS Nano.

[46] Zhang W, Ou JZ, Tang SY, Sivan V, Yao DD (2014). Liquid metal/metal oxide frameworks. Adv. Funct. Mater..

[47] Yang Y, Sun N, Wen Z, Cheng P, Zheng H (2018). Liquid-metal-based super-stretchable and structure-designable triboelectric nanogenerator for wearable electronics. ACS Nano.

[48] Zou H, Zhang Y, Guo L, Wang P, He X (2019). Quantifying the triboelectric series. Nat. Commun..

[49] Wang H, Chen X, Tian Z, Jiang Z, Yu W (2020). Efficient color manipulation of zinc sulfide-based mechanoluminescent elastomers for visualized sensing and anti-counterfeiting. J. Lumin..

[50] Li C, He Q, Wang Y, Wang Z, Wang Z (2022). Highly robust and soft biohybrid mechanoluminescence for optical signaling and illumination. Nat. Commun..

[51] Mao Y, Kubota Y, Gong J, Kurose T, Ishigam A (2021). Mechanical performance and visual fracture warning function of mechanochromic stimuli-recovery polymer networks. Macromolecules.

[52] Ding Y, So B, Cao J, Wondraczek L (2022). Ultrasound-induced mechanoluminescence and optical thermometry toward stimulus-responsive materials with simultaneous trigger response and read-out functions. Adv. Sci..

[53] Wang W, Li M, Zhou P, Yan Z, Wang D (2022). Design and synthesis of mechanochromic poly (ether-ester-urethane) elastomer with high toughness and resilience mediated by crystalline domains. Polym. Chem..

[54] Mao Y, Kubota Y, Kurose T, Ishigami A, Seshimo K (2020). Energy dissipation and mechanoresponsive color evaluation of a poly (n-hexyl methacrylate) soft material enhanced by a mechanochromic cross-linker with dynamic covalent bonds. Macromolecules.

[55] Chen B, Yang N, Jiang Q, Chen W, Yang Y (2018). Transparent triboelectric nanogenerator-induced high voltage pulsed electric field for a self-powered handheld printer. Nano Energy.

[56] Gao L, Chen X, Lu S, Zhou H, Xie W (2019). Enhancing the output performance of triboelectric nanogenerator via grating-electrode-enabled surface plasmon excitation. Adv. Energy Mater..

[57] Li R, Zhang H, Wang L, Liu G (2021). A contact-mode triboelectric nanogenerator for energy harvesting from marine pipe vibrations. Sensors.

[58] Chen X, Gao L, Chen J, Lu S, Zhou H (2020). A chaotic pendulum triboelectric-electromagnetic hybridized nanogenerator for wave energy scavenging and self-powered wireless sensing system. Nano Energy.

[59] Liu X, Shang Y, Zhang J, Zhang C (2021). Ionic liquid-assisted 3d printing of self-polarized beta-pvdf for flexible piezoelectric energy harvesting. ACS Appl. Mater. Interfaces.

[60] Ha M, Lim S, Cho S, Lee Y, Na S (2018). Skin-inspired hierarchical polymer architectures with gradient stiffness for spacer-free, ultrathin, and highly sensitive triboelectric sensors. ACS Nano.

[61] Jiang W (2020). Mnist-mix: a multi-language handwritten digit recognition dataset. IOP SciNotes.

[62] Madakannu A, Selvaraj A (2020). Digi-net: a deep convolutional neural network for multi-format digit recognition. Neural Comput. Appl..

[63] Dai C, Wang Y, Shan Y, Ye C, Lv Z (2023). Cytoskeleton-inspired hydrogel ionotronics for tactile perception and electroluminescent display in complex mechanical environments. Mater. Horiz..

[64] Dai C, Ye C, Ren J, Yang S, Cao L (2022). Humanoid ionotronic skin for smart object recognition and sorting. ACS Mater. Lett..

[65] Han Q, Gui C, Xu J, Lacidogna G (2019). A generalized method to predict the compressive strength of high-performance concrete by improved random forest algorithm. Constr. Build. Mater..

[66] Lang L, Tiancai L, Shan A, Xiangyan T (2021). An improved random forest algorithm and its application to wind pressure prediction. Int. J. Intell. Syst..

[67] Abellan J, Mantas CJ, Castellano JG, Moral-Garcia S (2018). Increasing diversity in random forest learning algorithm via imprecise probabilities. Expert Syst. Appl..

