# Supplementary material for: Mechanoluminescent-Triboelectric Bimodal Sensors for Self-Powered Sensing and Intelligent Control
Source: Nanomicro Lett. 2023 Mar 24;15:72. doi: 10.1007/s40820-023-01054-0 (PMC10039194; doi:10.1007/s40820-023-01054-0)
Supplement: Supplementary file 6 — Supplementary file6 (PDF 974 KB) [file 40820_2023_1054_MOESM6_ESM.pdf]

Supporting Information for

Mechanoluminescent-Triboelectric Bimodal Sensors for Self-Powered Sensing and Intelligent Control

Bo Zhou[1], Jize Liu[1], Xin Huang[1], Xiaoyan Qiu[1], Xin Yang[1], Hong Shao[2], Changyu Tang[2,]*, Xinxing Zhang[1,]*

[1]State Key Laboratory of Polymer Materials Engineering, Polymer Research Institute, Sichuan University, Chengdu 610065, P. R. China

[2]Chengdu Development Center of Science and Technology, China Academy of Engineering Physics, Chengdu 610200, P. R. China

*Corresponding authors. E-mail: xxzwwh@scu.edu.cn (X. Zhang); sugarchangyu@163.com (C. Tang)

Supplementary Figures

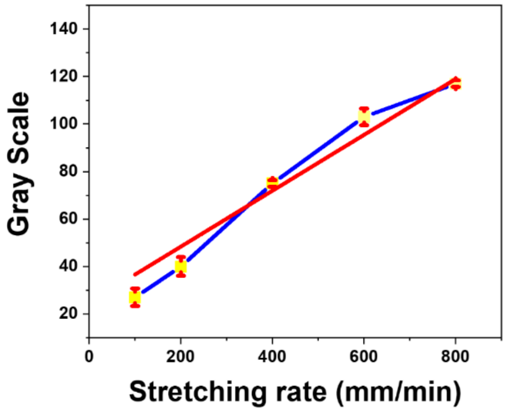

Fig. S1 Grayscale scale as a function of stretch rate

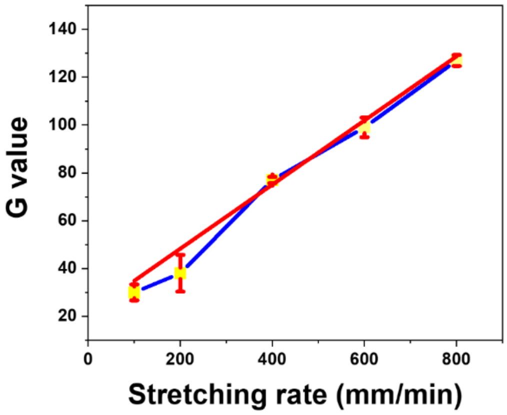

Fig. S2 G value as a function of stretch rate

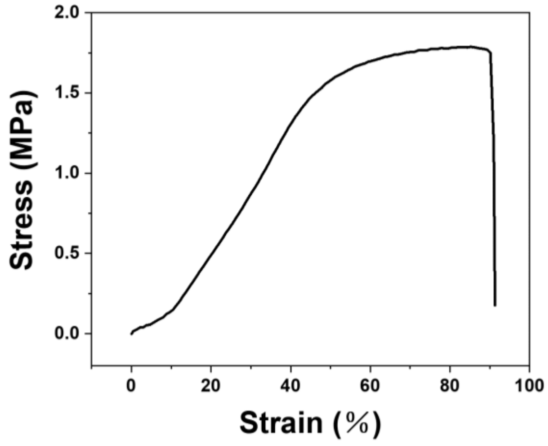

Fig. S3 Stress-strain curve of the composite elastomer

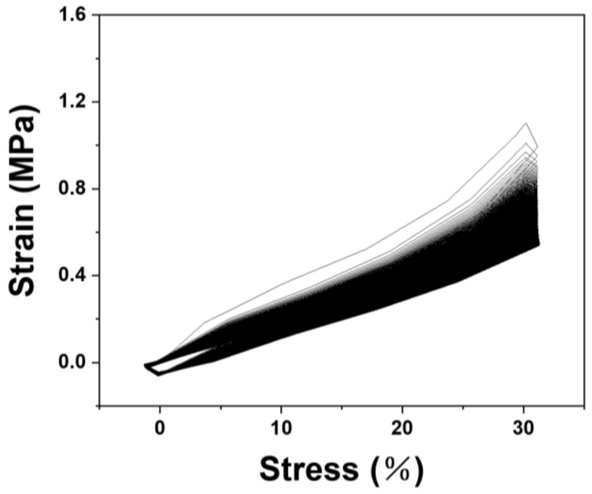

Fig. S4 Stress-strain curves for 2000 tensile cycles

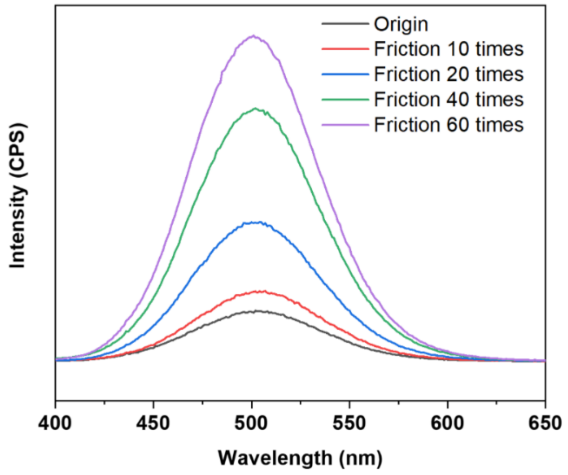

Fig. S5 Fluorescence spectra before and after friction with PET

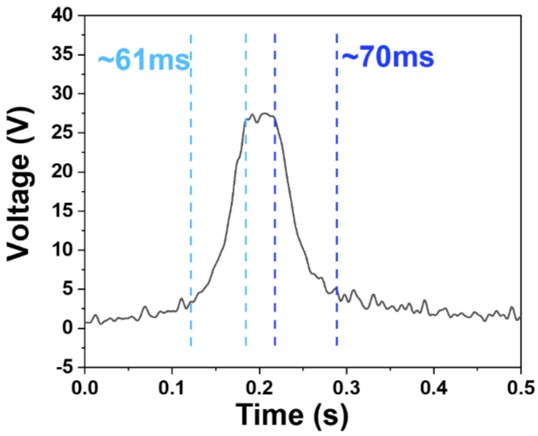

Fig. S6 Response time and recovery time of the STME

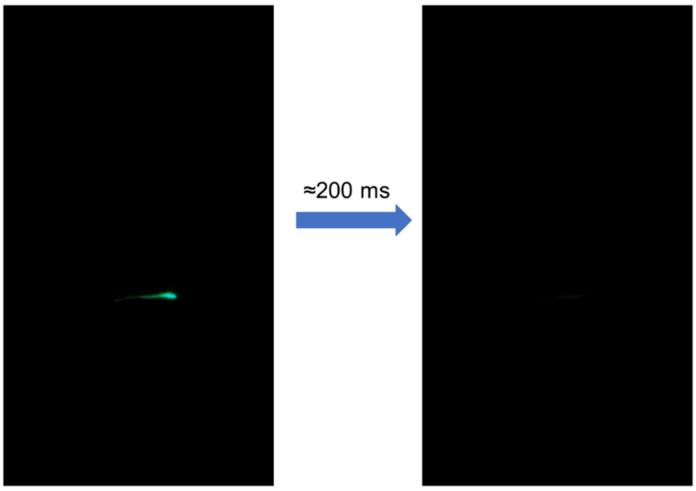

Fig. S7 Time of the ML

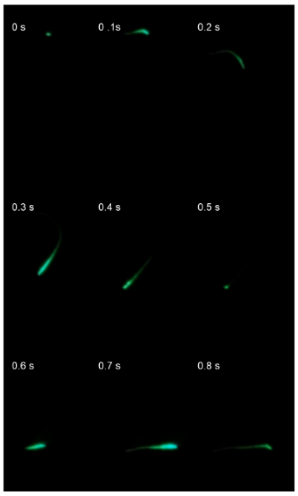

Fig. S8 Continuous photos taken with the number 2 written in real time

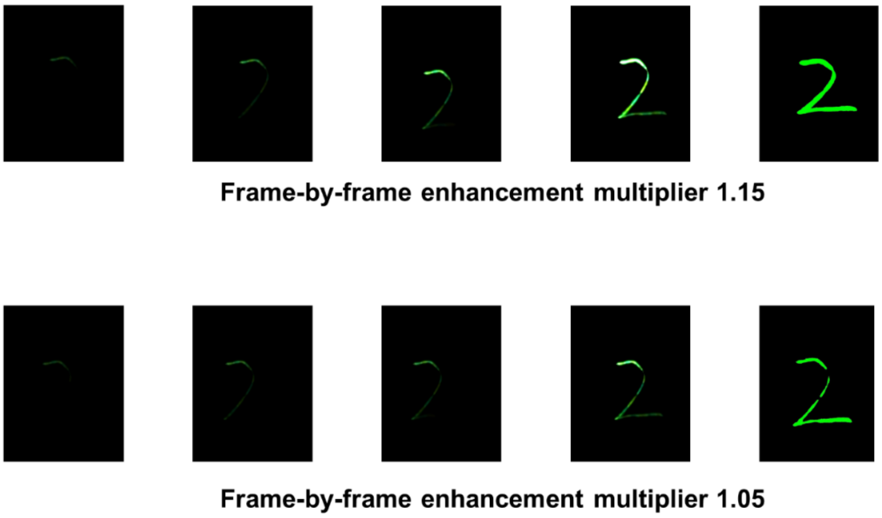

Fig. S9 Comparison of different frame-by-frame enhancement multiplier

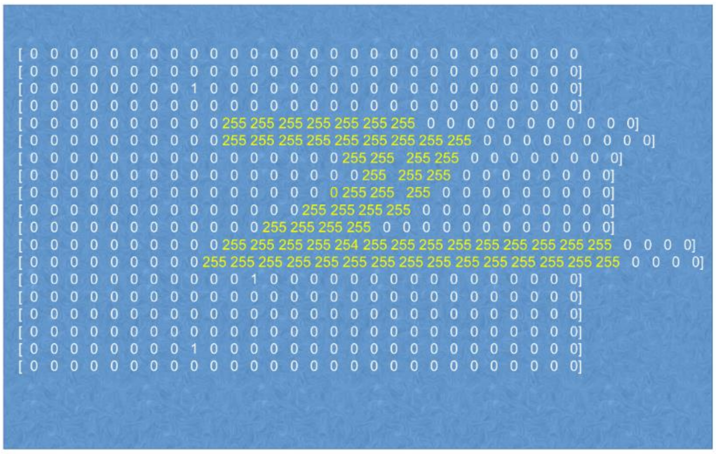

Fig. S10 Digitized matrix of handwritten Arabic numerals 2

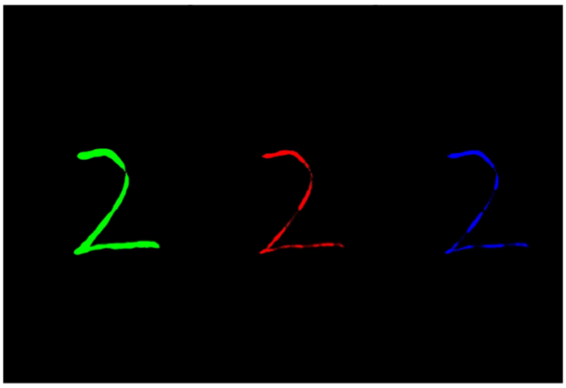

Fig. S11 Images of extracting red, green and blue colors respectively

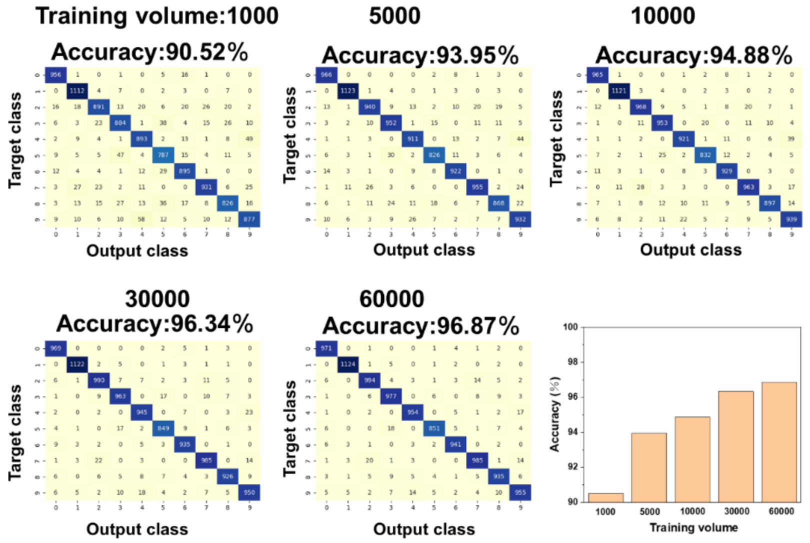

Fig. S12 Prediction confusion matrix using 1000, 5000, 10000, 30000, 60000 training volumes

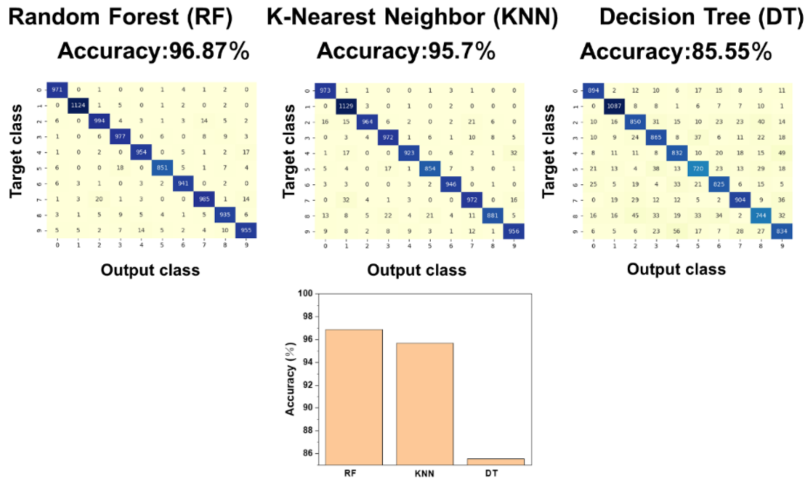

Fig. S13 Prediction confusion matrix using RF, KNN and DT algorithm

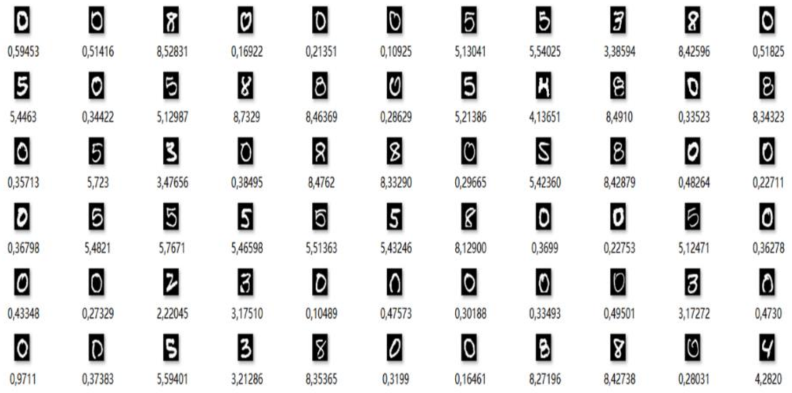

Fig. S14 Arabic numerals in an open source third-party handwritten numeral database
